# Expression of thymidylate synthase in gastric cancer patients treated with 5-fluorouracil and doxorubicin-based adjuvant chemotherapy after curative resection

**DOI:** 10.1054/bjoc.2000.1553

**Published:** 2001-01

**Authors:** J-H Choi, H-Y Lim, D K Nam, H S Kim, D Y Cho, J W Yi, H C Kim, Y K Cho, M W Kim, H J Joo, K B Lee, K B Kim

**Affiliations:** Departments of Hematology-Oncology, Surgery, Pathology, Ajou University School of Medicine, Suwon, 442-721, Korea; Division of Medicine, The University of Texas MD Anderson Cancer Center, Houston, TX, 77030, USA

**Keywords:** gastric cancer, thymidylate synthase, adjuvant chemotherapy, drug resistance, prognosis

## Abstract

We evaluated the expression of thymidylate synthase (TS) in locally advanced gastric cancer patients treated with adjuvant chemotherapy after curative resection and investigated the association between TS expression and clinicopathologic characteristics including prognosis of the patients. TS expression was evaluated by immunohistochemical staining using TS106 monoclonal antibody in 103 locally advanced gastric cancer patients (stage IB–IV) who underwent 5-fluorouracil (5-FU) and doxorubicin-based adjuvant chemotherapy after curative resection. 65 patients (63%) had primary tumours with high TS expression (≥ 25% of tumour cells positive), and 38 patients (37%) demonstrated low TS expression (< 25% of tumour cells positive or no staining). High TS expression was associated with male gender (*P* = 0.002), poorly differentiated histology (*P* = 0.015), and mixed type in Lauren’s classification (*P* = 0.027). There were no statistically significant differences in 4-year disease-free survival (60.0% vs 57.2%, *P* = 0.548) and overall survival (59.6% vs 59.3%, *P* = 0.792) between high-TS group and low-TS group. In conclusion, although high TS expression was associated with poorly differentiated histology and mixed type in Lauren's classification, it did not predict poor disease-free and overall survival in gastric cancer patients treated with 5-FU and doxorubicin-based adjuvant chemotherapy after curative resection. Further prospective studies including the evaluation of other biological markers associated with the resistance to 5-FU and doxorubicin are necessary. © 2001 Cancer Research Campaign http://www.bjcancer.com


British Journal of Cancer (2001) 84(2), 186–192
© 2001 Cancer Research Campaign
doi: 10.1054/ bjoc.2000.1553, available online at http://www.idealibrary.com on IDEAL®

http://www.bjcancer.com

### Expression of thymidylate synthase in gastric cancer patients treated with 5-fluorouracil and doxorubicin-based adjuvant chemotherapy after curative resection

**J-H Choi[1], H-Y Lim[1], DK Nam[1], HS Kim[1], DY Cho[1], JW Yi[1], HC Kim[1], YK Cho[2], MW Kim[2], HJ Joo[3], KB Lee[3] and KB Kim[4]**

Departments of [1]Hematology-Oncology, [2]Surgery and [3]Pathology, Ajou University School of Medicine, Suwon, 442-721, Korea; [4]Division of Medicine, The University of Texas MD Anderson Cancer Center, Houston, TX 77030, USA

**Summary** We evaluated the expression of thymidylate synthase (TS) in locally advanced gastric cancer patients treated with adjuvant chemotherapy after curative resection and investigated the association between TS expression and clinicopathologic characteristics including prognosis of the patients. TS expression was evaluated by immunohistochemical staining using TS106 monoclonal antibody in 103 locally advanced gastric cancer patients (stage IB–IV) who underwent 5-fluorouracil (5-FU) and doxorubicin-based adjuvant chemotherapy after curative resection. 65 patients (63%) had primary tumours with high TS expression (≥ 25% of tumour cells positive), and 38 patients (37%) demonstrated low TS expression (< 25% of tumour cells positive or no staining). High TS expression was associated with male gender ($P$ = 0.002), poorly differentiated histology ($P$ = 0.015), and mixed type in Lauren's classification ($P$ = 0.027). There were no statistically significant differences in 4-year disease-free survival (60.0% vs 57.2%, $P$ = 0.548) and overall survival (59.6% vs 59.3%, $P$ = 0.792) between high-TS group and low-TS group. In conclusion, although high TS expression was associated with poorly differentiated histology and mixed type in Lauren's classification, it did not predict poor disease-free and overall survival in gastric cancer patients treated with 5-FU and doxorubicin-based adjuvant chemotherapy after curative resection. Further prospective studies including the evaluation of other biological markers associated with the resistance to 5-FU and doxorubicin are necessary. © 2001 Cancer Research Campaign http://www.bjcancer.com

**Keywords**: gastric cancer; thymidylate synthase; adjuvant chemotherapy; drug resistance; prognosis

Although the incidence of gastric cancer has been declining in the Western world, it is the most common malignancy in many other countries including Korea (Kim et al, 1989; Fuchs and Mayer, 1995). Curative surgery is an essential treatment for the long-term survival of patients with gastric cancer (Noguchi et al, 1989; Shiu et al, 1989; Fuchs and Mayer, 1995). However, the prognosis of patients with locally advanced gastric cancer is generally poor even with curative resection (Noguchi et al, 1989; Shiu et al, 1989; Fuchs and Mayer, 1995). Adjuvant chemotherapy has failed to demonstrate a significant survival benefit for such patients in most randomized trials and meta-analyses, except in a few studies with positive results (Shiu et al, 1989; Krook et al, 1991; Kim et al, 1992; Hermans et al, 1993; Nakazato et al, 1994; Fuchs and Mayer, 1995; Lise et al, 1995; Macdonald et al, 1995; Kelsen, 1996; Kim et al, 1998; Cirera et al, 1999; Earle and Maroun, 1999).

In adjuvant chemotherapy of gastric cancer, 5-fluorouracil (5-FU) has been considered as one of the standard chemotherapeutic agents and is usually combined with other agents such as doxorubicin and mitomycin-C (Krook et al, 1991; Kim et al, 1992; Nakazato et al, 1994; Lise et al, 1995; Macdonald et al, 1995; Kelsen, 1996; Kim et al, 1998). Thymidylate synthase (TS) catalyzes the methylation of deoxyuridine monophosphate to deoxythymidine monophosphate, which is an essential process for DNA synthesis (Pinedo and Peters, 1988). TS is also a critical target of 5-FU. In tumour cells, 5-FU is converted to 5-fluorodeoxyuridine monophosphate, which forms a tight-binding covalent complex with TS in the presence of folate cofactor 5, 10-methylene tetrahydrofolate (Pinedo and Peters, 1988). Several preclincal and clinical studies have demonstrated that increased expression of TS may be associated with 5-FU resistance in a variety of malignancies (Washtien, 1984; Johnston et al, 1992; Johnston et al., 1995; Lenz et al, 1996; Boku et al, 1998; Yeh, et al, 1998).

In gastric cancer, several studies have shown that TS expression has an inverse relationship with response to chemotherapy and survival in patients with locally advanced or metastatic disease receiving systemic chemotherapy with 5-FU-containing regimens (Johnston et al, 1995; Lenz et al, 1996; Boku et al, 1998; Yeh, et al, 1998). Moreover, TS expression also predicted poor disease-free and overall survival in a few reports that investigated gastric cancer patients treated with 5-FU-based adjuvant chemotherapy after surgical resection (Kuniyasu et al, 1998; Suda et al, 1999). We evaluated the expression of TS using immunohistochemistry in primary tumours of locally advanced gastric cancer patients treated with 5-FU and doxorubicin-based adjuvant chemotherapy after curative resection and investigated the association between TS expression and various clinicopathologic characteristics including prognosis of the patients.

*Received 10 February 2000*
*Revised 21 August 2000*
*Accepted 13 September 2000*

*Correspondence to:* J-H Choi. E-mail: jhchoimj@unitel.co.kr

### MATERIALS AND METHODS

### Patients

Included in this study were 103 patients with locally advanced gastric adenocarcinoma who underwent 5-FU and doxorubicinbased adjuvant chemotherapy after curative surgical resection at Ajou University Medical Center in Suwon, Korea, between July 1994 and October 1996. All patients had a postsurgical pathologic stage ranging from IB to IV without evidence of distant metastasis according to the American Joint Committee on Cancer TNM classification (American Joint Committee on Cancer, 1997). Curative resection was defined by the General Rules for the Gastric Cancer Study in Surgery and Pathology of the Japanese Research Society for Gastric Cancer as: no involvement of surgical stumps; sufficient lymphatic dissection (R number ≥ N number); no distant metastasis; removal of involved adjacent organs and structures by combined en-bloc resection; and no gross residual disease (Japanese Research Society for Gastric Cancer, 1981). None of these patients received chemotherapy prior to surgery.

Although all patients received 5-FU and doxorubicin-based adjuvant chemotherapy, the regimens were not uniform. The most commonly administered regimen was FA (5-FU, doxorubicin) with OK-432 (51 patients), followed by FAM (5-FU, doxorubicin, mitomycin-C) (33 patients), FA (11 patients), and FA with lentinan (eight patients). Chemotherapy was usually started 2–3 weeks after surgery. In the FA regimen with or without immunotherapy (OK-432 or lentinan), 5-FU (500 mg $m^{-2}$ $day^{-1}$) was given by intravenous (iv) infusion for 30 min on day 1, 8, 15, and doxorubicin (40 mg $m^{-2}$) was given by rapid iv injection on day 1. The treatment was repeated every 3 weeks for 12 cycles. OK-432 (2.0 Klinishe Einheit $day^{-1}$) and lentinan (2 mg $day^{-1}$) were administered intramuscularly and intravenously, respectively, weekly throughout the FA chemotherapy period. In the FAM regimen, 5-FU (1000 mg $m^{-2}$ $day^{-1}$) was given by continuous iv infusion on days 1–3, and doxorubicin (40 mg $m^{-2}$) and mitomycin-C (10 mg $m^{-2}$, every other cycle) were administered by rapid iv injection on day 1. The treatment was repeated every 4 weeks for 12 cycles and mitomycin-C was administered every 8 weeks. Patients were evaluated weekly during the treatment period and every 3 months after completion of chemotherapy. Follow-up studies were performed every 6 months: blood cell count and blood chemistry, chest X-ray, abdominal ultrasonograpy or computed tomography, and upper gastrointestinal series or esophagogastroduodenoscopy.

### Immunohistochemical staining

Formalin-fixed, paraffin-embedded tissue sections of 4 μM thickness were deparaffinized in xylene and rehydrated through graded alcohols. After microwave pretreatment in citrate buffer (pH 6.0) for antigen retrieval, slides were immersed in 0.3% hydrogen peroxide for 20 min to block the endogenous peroxidase activity. After washing, slides were incubated overnight at 4˚C with the TS 106 mouse monoclonal antibody against TS (Neomarkers, Fremont, CA, USA) at a dilution of 1:100. The slides were then stained by the avidin–biotin-complex (ABC) peroxidase method with commercially available UltraTech HRP kit (Immunotech, Marseilles, France) according to the instructions of the manufacturer. Reaction products were visualized by immersing slides in diaminobenzidine tetrahydrochloride. Finally, the slides were counterstained with Mayer's haematoxylin and mounted with glass coverslips.

Formalin-fixed, paraffin-embedded sections of colon adenocarcinoma known to have high expression of TS were used as positive controls. The negative controls were made by the omission of the primary antibody during the process of immunohistochemical staining.

### Tissue evaluation

The slides were examined independently by two observers (HJJ and KBL) blinded to both clinical and pathologic data. TS expression was quantified using a visual grading system based on the extent of staining, focal pattern (< 25% of tumour cells positive or no staining) or diffuse pattern (≥25% of tumour cells positive) as well as the intensity of staining (0–3+) (Figure 1). There was close agreement (> 90%) in the TS evaluation between both investigators. In case of disagreement, final grading was determined by consensus. The expression of TS was divided into high-TS (diffuse pattern) and low-TS (focal pattern) groups according to the extent of staining.

### Statistical analysis

Disease-free survival and overall survival were calculated using the Kaplan–Meier method (Kaplan and Meier, 1958). Disease-free survival was defined as the time from the day of operation to a documented recurrence, or second primary cancer, or death from any other cause. Data on patients who did not have a recurrence were censored at the last follow-up. Overall survival was defined as the time from the day of operation to death; data on survivors were censored at the last follow-up. The differences between the survival curves were tested by using the log-rank test. Comparison of variables according to TS expression was evaluated with the Student's *t*-test and chi-square test.

### RESULTS

### Patient characteristics

Of 103 patients who were assessable by TS staining, 71 were male and 32 were female, and their median age was 53 years (range 28–72). Stages were IB in nine, II in 28, IIIA in 31, IIIB in 17, and IV in 18 patients. The median number of chemotherapy cycles was eight (range 1–13).

### Association of TS expression with patient and tumour characteristics

TS staining pattern in gastric tumours showed predominantly a granular cytoplasmic staining in tumour cells (Figure 1). In some tumor tissues, positive nuclear staining for TS was present in addition to cytoplasmic staining (Figure 1). Within the study group, 65 patients (63%) had primary tumours with high TS expression (≥ 25% of tumour cells positive), and 38 patients (37%) demonstrated low TS expression (< 25% of tumour cells positive or no staining). 78% of the tumours showed high intensity of staining (intensity 2+ or 3+), whereas 22% had low intensity of staining (intensity 0 or 1+). All tumours with diffuse pattern (≥ 25% of tumour cells positive) in the extent of staining demonstrated high intensity of staining (intensity 2+ or 3+).

The proportion of male was higher in high-TS group (80%) compared with low-TS group (50%) ($P$ = 0.002). In tumour differentiation, poorly differentiated histology was more common in the high-TS group (43%) than in the low-TS group (24%) ($P$ = 0.015). TS expression was also associated with Lauren's classification ($P$ = 0.027). Mixed-type tumours were more frequently observed in the high-TS group (40%) compared with the low-TS group (21%). There was no significant association between TS

© 2001 Cancer Research Campaign

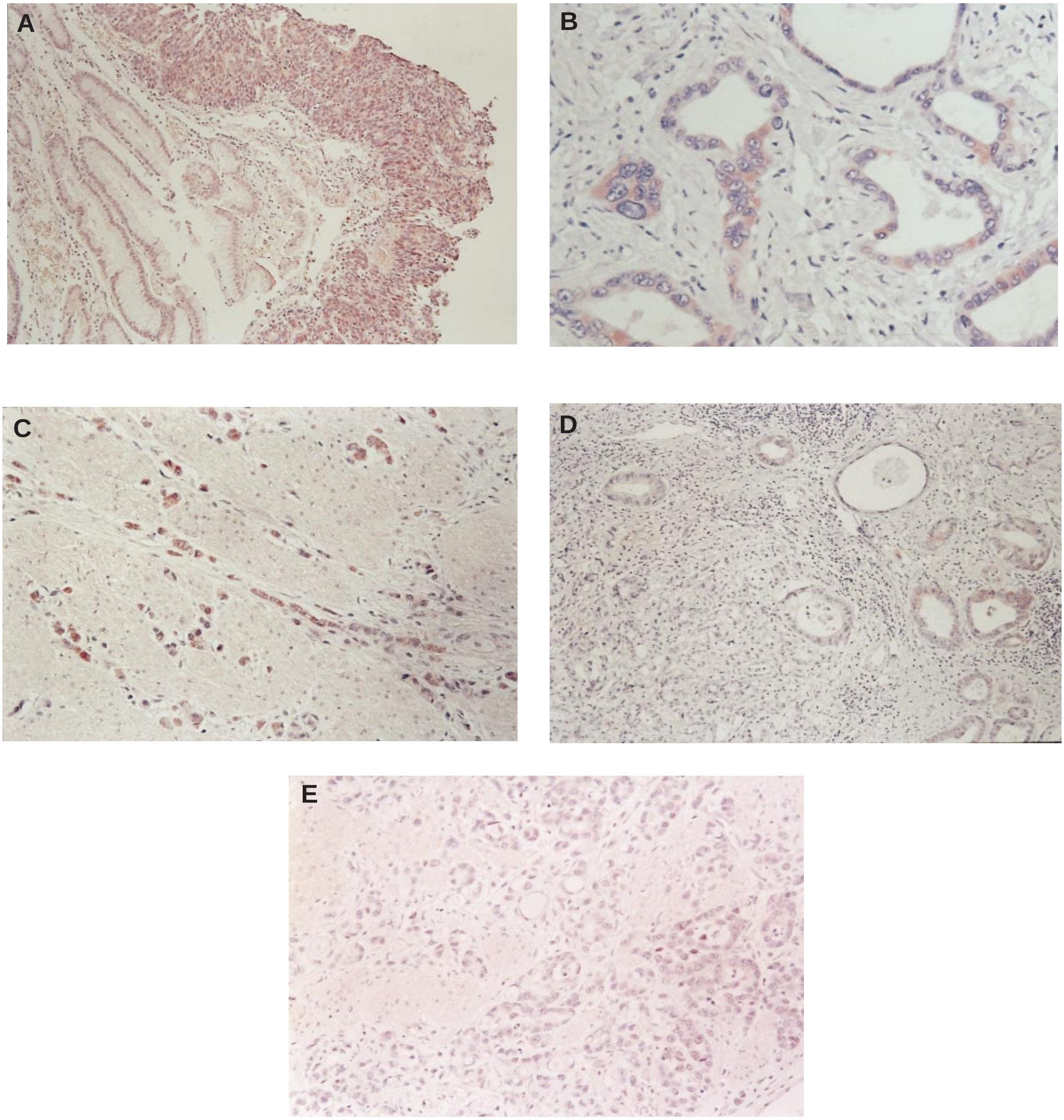

**Figure 1** Immunohistochemical staining of thymidylate synthase (TS) in gastric cancer. (**A**) Diffuse, high intensity (3+) TS staining in both cytoplasm and nucleus of gastric cancer cells in contrast with no immunoreactivity in normal gastric mucosa (× 100). (**B**) Diffuse, high intensity (3+) TS staining in well differentiated tubular adenocarcinoma (× 200). (**C**) Diffuse, high intensity (3+) TS staining in signet-ring cell carcinoma (× 200). (**D**) Focal, high intensity (3+) TS staining in well differentiated adenocarcinoma area in contrast with negative immunostaining in moderately to poorly differentiated adenocarcinoma area (× 100). (**E**) Focal, low intensity (1+) TS staining in moderately differentiated tubular adenocarcinoma (× 200)

expression and other patient and tumour characteristics including age, type of operation, primary tumour size, location of tumour, depth of tumour invasion, lymph node metastasis, postoperative pathologic stage, and regimens of chemotherapy (Table 1).

### Association of TS expression with outcome of patients

The median follow-up duration of the survivors was 51 months (range 36–63) and no patient was lost to follow-up. At the time of

 *© 2001 Cancer Research Campaign*

**Table 1** Characteristics of patients according to TS expression[a]

| Characteristics | Low TS | High TS | *P* value |
|---|---|---|---|
| Age (years) | 50.6 ± 9.8 | 53.1 ± 9.3 | 0.195 |
| Gender | | | 0.002 |
| Male | 19 (50) | 52 (80) | |
| Female | 19 (50) | 13 (20) | |
| Type of operation | | | 0.410 |
| Subtotal | 27 (71) | 41 (63) | |
| Total | 11 (29) | 24 (37) | |
| Primary tumour size (cm) | 6.2 ± 3.4 | 5.7 ± 2.4 | 0.332 |
| Tumour location | | | 0.086 |
| Lower | 21 (55) | 34 (52) | |
| Mid | 9 (24) | 24 (37) | |
| Upper | 5 (13) | 7 (11) | |
| Diffuse | 3 (8) | 0 (0) | |
| Borrmann type | | | 0.403 |
| I | 1 (3) | 5 (8) | |
| II | 11 (29) | 12 (19) | |
| III | 21 (55) | 42 (65) | |
| IV | 5 (13) | 6 (9) | |
| Tumour differentiation | | | 0.015 |
| Well | 3 (8) | 10 (15) | |
| Moderate | 12 (32) | 19 (29) | |
| Poor | 9 (24) | 28 (43) | |
| Signet-ring | 9 (24) | 8 (12) | |
| Undifferentiated | 1 (3) | 0 (0) | |
| Mucinous | 4 (10) | 0 (0) | |
| Lauren's classification | | | 0.027 |
| Intestinal | 22 (58) | 35 (54) | |
| Diffuse | 8 (21) | 4 (6) | |
| Mixed | 8 (21) | 26 (40) | |
| Lymphatic invasion | | | 0.915 |
| Yes | 29 (76) | 49 (75) | |
| No | 9 (24) | 16 (25) | |
| Depth of tumour invasion | | | 0.368 |
| T2 | 17 (45) | 25 (39) | |
| T3 | 21 (55) | 37 (57) | |
| T4 | 0 (0) | 3 (5) | |
| Lymph node involvement | | | 0.698 |
| N0 | 7 (18) | 11 (17) | |
| N1 | 15 (40) | 26 (40) | |
| N2 | 12 (32) | 16 (25) | |
| N3 | 4 (10) | 12 (19) | |
| Stage | | | 0.415 |
| IB | 4 (10) | 5 (8) | |
| II | 9 (24) | 19 (29) | |
| IIIA | 15 (40) | 16 (25) | |
| IIIB | 6 (16) | 11 (17) | |
| IV | 4 (10) | 14 (21) | |
| Chemotherapy | | | 0.396 |
| FA + OK-432[b] | 21 (55) | 30 (46) | |
| FAM[b] | 11 (29) | 22 (34) | |
| FA | 5 (13) | 6 (9) | |
| FA + lentinan | 1 (3) | 7 (11) | |
| Cycles of chemotherapy | 8.0 ± 4.0 | 8.8 ± 3.3 | 0.243 |

[a]Values are number or mean ± standard deviation, with percentages in parentheses; [b]F, 5-FU, A, doxorubicin, M, mitomycin-C.

analysis, 38 patients had recurrences, and two had second primary cancer (hepatocellular carcinoma and laryngeal cancer). 42 of the 103 patients have died. Death due to recurrence of gastric cancer occurred in 37 patients. One patient died of gastrointestinal bleeding without evidence of recurrence, one patient died of second primary cancer (hepatocellular carcinoma), and the causes of death were undetermined in three patients. There was no statistically significant difference in 4-year disease-free survival between high-TS group and low-TS group (60.0% vs 57.2%, $P = 0.548$) (Figure 2). Four-year overall survival also demonstrated no significant difference between the two groups (59.6% vs 59.3%, $P = 0.792$) (Figure 3).

In the intensity of TS staining, no differences in disease-free survival ($P = 0.420$) and overall survival of the patients ($P = 0.769$) were noted between high-intensity group and low-intensity group. There were no significant differences in disease-free survival and overall survival according to other clinicopathologic characteristics except primary tumour location (Table 2).

### DISCUSSION

Both 5-FU and doxorubicin are effective agents in the chemotherapy of gastric cancer in the adjuvant setting, as well as in

© 2001 Cancer Research Campaign 

**Table 2** Disease-free and overall survival of the patients according to various characteristics

| Characteristics | Patients (*n*) | 4-year disease-free survival (%) | *P* value | 4-year overall survival (%) | *P* value |
|---|---|---|---|---|---|
| Primary tumour size | | | 0.053 | | 0.056 |
| ≤ 5 cm | 52 | 69.1 | | 68.5 | |
| > 5 cm | 51 | 48.2 | | 50.1 | |
| Tumour location | | | 0.022 | | 0.029 |
| Lower | 55 | 65.1 | | 67.2 | |
| Mid | 33 | 54.6 | | 52.4 | |
| Upper | 12 | 58.3 | | 57.1 | |
| Diffuse | 3 | 0.0 | | 0.0 | |
| Tumour differentiation | | | 0.667 | | 0.717 |
| Well | 13 | 69.2 | | 68.4 | |
| Moderate | 31 | 58.1 | | 58.1 | |
| Poor or undifferentiated | 38 | 57.3 | | 59.2 | |
| Signet-ring | 17 | 52.9 | | 52.3 | |
| Mucinous | 4 | 75.0 | | 66.7 | |
| Lauren's classification | | | 0.946 | | 0.901 |
| Intestinal | 57 | 59.7 | | 58.3 | |
| Diffuse | 12 | 58.3 | | 58.3 | |
| Mixed | 34 | 58.0 | | 61.1 | |
| Lymphatic invasion | | | 0.430 | | 0.307 |
| Yes | 78 | 55.9 | | 56.6 | |
| No | 25 | 68.0 | | 68.0 | |
| Depth of tumour invasion | | | 0.309 | | 0.452 |
| T2 | 42 | 69.1 | | 67.6 | |
| T3 | 58 | 53.0 | | 54.8 | |
| T4 | 3 | 33.3 | | 33.3 | |
| Lymph node involvement | | | 0.524 | | 0.407 |
| N0 | 18 | 77.8 | | 77.8 | |
| N1 | 41 | 60.8 | | 59.8 | |
| N2 | 28 | 49.5 | | 53.1 | |
| N3 | 16 | 50.0 | | 48.2 | |
| Stage | | | 0.419 | | 0.462 |
| IB | 9 | 77.8 | | 77.8 | |
| II | 28 | 71.4 | | 70.6 | |
| IIIA | 31 | 57.9 | | 57.1 | |
| IIIB | 17 | 40.3 | | 47.1 | |
| IV | 18 | 50.0 | | 48.1 | |
| Treatment | | | 0.866 | | 0.781 |
| Chemotherapy + immunotherapy | 59 | 59.3 | | 59.2 | |
| Chemotherapy | 44 | 57.5 | | 60.0 | |
| Chemotherapy regimens | | | 0.671 | | 0.802 |
| FA ± immunotherapy | 70 | 61.4 | | 61.2 | |
| FAM[a] | 33 | 51.2 | | 55.4 | |

[a]F, 5-FU; A, doxorubicin; M, mitomycin-C.

unresectable or metastatic disease (Kim et al, 1992; Nakazato et al, 1994; Kelsen, 1996; Wils, 1996; Kim et al, 1998). Biological markers, which can predict the resistance of tumours to these agents, may provide valuable information in the treatment of gastric cancer with systemic chemotherapy. In this study, we evaluated the clinical significance of TS expression as a prognostic marker of recurrence and survival in locally advanced gastric cancer patients treated with 5-FU and doxorubicin-based adjuvant chemotherapy after curative resection.

In terms of TS expression in primary tumours, 63% of tumours demonstrated high expression of TS. The proportion of the patients with high TS expression in the current study seems to be high compared to other reports that evaluated the TS expression with surgically resected gastric cancer tissues by immunohistochemistry. Kuniyasu et al (1998) and Suda et al (1999) demonstrated that high expression of TS was observed in 42% and 46% of cases, respectively. This may be explained by differences in the antibodies used for TS immunostaining and in the stage distribution of the patients. In the study by Kuniyasu et al (1998), 28% of patients had early gastric cancer, and only stage IIIB patients were included in the study by Suda et al (1999).

In the current study, high TS expression was associated with poorly differentiated histology in tumour grade and mixed type in Lauren's classification. While the prognostic significance of poorly differentiated histology in gastric cancer is controversial, several studies have demonstrated that mixed type in Lauren's classification is associated with poor prognosis and other factors suggesting aggressive biologic behavior such as lymph node metastasis and advanced stage (Shiu et al, 1989; Kim et al, 1992; Carneiro et al, 1995; Macdonald et al, 1995; Yu et al, 1995; Setälä et al, 1996; Stelzner and Emmrich, 1997). Unlike the present results, other studies with gastric cancer tissue have failed to show any relationship between TS expression and tumour differentiation or Lauren's classification (Kuniyasu et al, 1998; Yeh et al, 1998; Suda et al, 1999). Considering the fact that mixed-type gastric cancer is associated with poor outcome of the patients, high TS expression may suggest intrinsic aggressive tumour behaviour.

 *© 2001 Cancer Research Campaign*

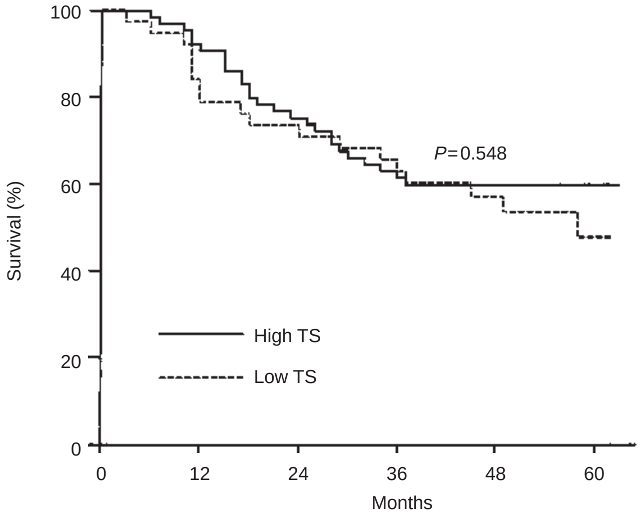

**Figure 2** Disease-free survival of gastric cancer patients according to TS expression

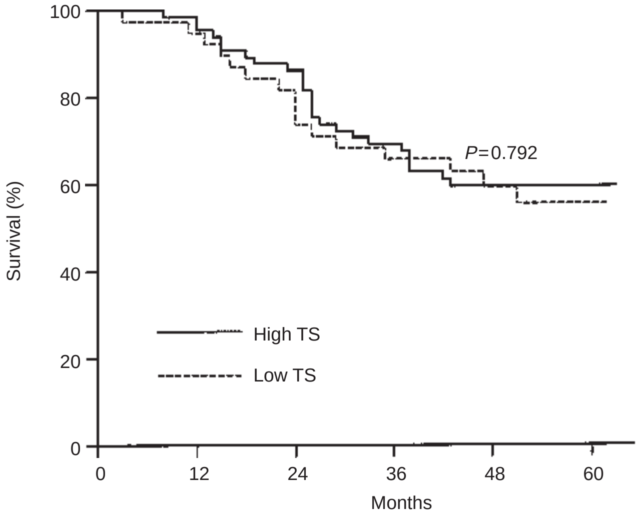

**Figure 3** Overall survival of gastric cancer patients according to TS expression

High TS expression was associated with a high proportion of male gender in the current study. It was an unexpected finding considering the previous reports, and further studies would be necessary to find a possible explanation for such a result (Kuniyasu et al, 1998; Yeh et al, 1998; Suda et al, 1999). TS expression was not associated with other characteristics of prognostic significance such as primary tumour size, depth of tumour invasion, lymph node metastasis and postoperative pathologic stage. Kuniyasu et al (1998) demonstrated a significant correlation between high TS expression and advanced stage. However, a significant proportion of the patients in their study had early gastric cancer, which makes comparison with our results difficult.

In gastric cancer, TS expression predicted increased risk of recurrence and poor survival in patients treated with adjuvant chemotherapy after surgical resection in a few studies (Kuniyasu et al, 1998; Suda et al, 1999). However, in the current study, there were no significant differences in disease-free survival and overall survival between the high-TS group and low-TS group. These results might have relevance to our findings of the lack of association between TS expression and important prognostic factors such as pathologic stage. The discrepant findings between the current study and the previous reports in terms of prognostic implications of TS could be explained as follows. First, the TS expression in primary tumours of gastric cancer patients may not reflect the ability of the tumour to induce TS protein or undergo TS gene amplification with exposure to chemotherapeutic agents (Johnston et al, 1994; Pestalozzi et al, 1997). Secondly, all patients in this study were treated with doxorubicin as well as 5-FU, while other studies demonstrating increased risk of recurrence and poor survival in gastric cancer patients with high TS expression used 5-FU or tegafur-uracil with or without mitomycin-C (Kuniyasu et al, 1998; Suda et al, 1999). Therefore, doxorubicin might have played a role in the failure of high TS expression to predict poor survival of patients. Thirdly, it is also possible that adjuvant chemotherapy did not affect survival regardless of TS expression, which may explain the lack of difference in survival between high-TS and low-TS groups in the current study. Alternatively, there is a possibility that the gastric cancer patients with high TS expression might experience a greater survival benefit from adjuvant chemotherapy compared to patients with low TS expression, as shown in other studies with rectal cancer and breast cancer (Johnston et al, 1994; Pestalozzi et al, 1997). In the current study, there was no significant difference in survival according to important prognostic variables such as Lauren's classification, lymph node metastasis and pathologic stage. These results suggest the possibility that the prognostic significance of various prognostic factors including TS expression may disappear with the use of adjuvant chemotherapy, bringing the survival curves closer. However, a larger study including patients treated with surgical resection alone would be essential to prove the latter two speculations.

In conclusion, high TS expression did not predict poor disease-free and overall survival in patients with locally advanced gastric cancer treated with 5-FU and doxorubicin-based chemotherapy after curative resection, although it was associated with poorly differentiated histology and mixed type of tumour in Lauren's classification. Further prospective study with a large number of patients, including the evaluation of other biological markers associated with the resistance to 5-FU and doxorubicin such as thymidine kinase, P-glycoprotein, multidrug resistance-associated protein, and topoisomerase II, would seem to be necessary.

### ACKNOWLEDGEMENTS

The authors are grateful to Ms Geum Sook Jeong for secretarial assistance.

### REFERENCES

American Joint Committee on Cancer (1997) *AJCC cancer staging manual, 5th edn*, pp. 71–76. Lippincott-Raven: Philadelphia

Boku N, Chin K, Hosokawa K, Ohtsu A, Tajiri H, Yoshida S, Yamao T, Kondo H, Shirao K, Shimada Y, Saito D, Hasebe T, Mukai K, Seki S, Saito H and Johnston PG (1998) Biological markers as a predictor for response and prognosis of unresectable gastric cancer patients treated with 5-fluorouracil and *cis*-platinum. *Clin Cancer Res* **4**: 1469–1474

Carneiro F, Seixas M and Sobrinho-Simoes M (1995) New elements for an updated classification of the carcinomas of the stomach. *Path Res Pract* **191**: 571–584

Cirera L, Balil A, Batiste-Alentorn E, Tusquets I, Cardona T, Arcusa A, Jolis L, Saigi E, Guasch I, Badia A and Boleda M (1999) Randomized clinical trial of adjuvant mitomycin plus tegafur in patients with resected stage III gastric cancer. *J Clin Oncol* **17**: 3810–3815

Earle CC and Maroun JA (1999) Adjuvant chemotherapy after curative resection for gastric cancer in non-Asian patients: revisiting a meta-analysis of randomised trials. *Eur J Cancer* **35**: 1059–1064
Fuchs CS and Mayer RJ (1995) Gastric carcinoma. *N Engl J Med* **333**: 32–41
Hermans J, Bonenkamp JJ, Boon MC, Bunt AMG, Ohyama S, Sasako M and Van de Velde CJH (1993) Adjuvant therapy after curative resection for gastric cancer: meta-analysis of randomized trials. *J Clin Oncol* **11**: 1441–1447
Japanese Research Society for Gastric Cancer (1981) The general rules for the gastric cancer study in surgery and pathology. *Jpn J Surg* **11**: 127–139
Johnston PG, Drake JC, Trepel J and Allegra CJ (1992) Immunological quantitation of thymidylate synthase using the monoclonal antibody TS 106 in 5-fluorouracil-sensitive and -resistant human cancer cell lines. *Cancer Res* **52**: 4306–4312
Johnston PG, Fisher ER, Rockette HE, Fisher B, Wolmark N, Drake JC, Chabner BA and Allegra CJ (1994) The role of thymidylate synthase expression in prognosis and outcome of adjuvant chemotherapy in patients with rectal cancer. *J Clin Oncol* **12**: 2640–2647
Johnston PG, Lenz H-J, Leichman CG, Danenberg KD, Allegra CJ, Danenberg PV and Leichman L (1995) Thymidylate synthase gene and protein expression correlate and are associated with response to 5-fluorouracil in human colorectal and gastric tumors. *Cancer Res* **55**: 1407–1412
Kaplan EL and Meier P (1958) Non-parametric estimation from incomplete observations. *J Am Statist Assoc* **53**: 457–481
Kelsen DP (1996) Adjuvant and neoadjuvant therapy for gastric cancer. *Semin Oncol* **23**: 379–389
Kim IS, Suh I, Oh HC, Kim BS and Lee Y (1989) Incidence and survival of cancer in Kangwha County (1983–1987). *Yonsei Med J* **30**: 256–268
Kim J-P, Kwon OJ, Oh ST and Yang HK (1992) Results of surgery on 6589 gastric cancer patients and immunochemosurgery as the best treatment of advanced gastric cancer. *Ann Surg* **216**: 269–279
Kim S-Y, Park HC, Yoon C, Yoon HJ, Choi YM and Cho KS (1998) OK-432 and 5-fluorouracil, doxorubicin, and mitomycin C (FAM-P) versus FAM chemotherapy in patients with curatively resected gastric carcinoma: a randomized phase III trial. *Cancer* **83**: 2054–2059
Krook JE, O'Connell MJ, Wieand HS, Beart RW Jr, Leigh JE, Kugler JW, Foley JF, Pfeifle DM and Twito DI (1991) A prospective, randomized evaluation of intensive-course 5-fluorouracil plus doxorubicin as surgical adjuvant chemotherapy for resected gastric cancer. *Cancer* **67**: 2454–2458
Kuniyasu T, Nakamura T, Tabuchi Y and Kuroda Y (1998) Immunohistochemical evaluation of thymidylate synthase in gastric carcinoma using a new polyclonal antibody: the clinical role of thymidylate synthase as a prognostic indicator and its therapeutic usefulness. *Cancer* **83**: 1300–1306
Lenz H-J, Leichman CG, Danenberg KD, Danenberg PV, Groshen S, Cohen H, Laine L, Crookes P, Silberman H, Baranda J, Garcia Y, Li J and Leichman L (1996) Thymidylate synthase mRNA level in adenocarcinoma of the stomach: a predictor for primary tumor response and overall survival. *J Clin Oncol* **14**: 176–182
Lise M, Nitti D, Marchet A, Sahmoud T, Buyse M, Duez N, Fiorentino M, Santos JGD, Labianca R, Rougier P and Gignoux M (1995) Final results of a phase III clinical trial of adjuvant chemotherapy with the modified fluorouracil, doxorubicin, and mitomycin regimen in resectable gastric cancer. *J Clin Oncol* **13**: 2757–2763
Macdonald JS, Fleming TR, Peterson RF, Berenberg JL, McClure S, Chapman RA, Eyre HJ, Solanki D, Cruz AB Jr, Gagliano R, Estes NC, Tangen CM and Rivkin S (1995) Adjuvant chemotherapy with 5-FU, adriamycin, and mitomycin-C (FAM) versus surgery alone for patients with locally advanced gastric adenocarcinoma: a Southwest Oncology Group study. *Ann Surg Oncol* **2**: 488–494
Nakazato H, Koike A, Saji S, Ogawa N and Sakamoto J (1994) Efficacy of immunochemotherapy as adjuvant treatment after curative resection of gastric cancer. *Lancet* **343**: 1122–1126
Noguchi Y, Imada T, Matsumoto A, Coit DG and Brennan MF (1989) Radical surgery for gastric cancer: a review of the Japanese experience. *Cancer* **64**: 2053–2062
Pestalozzi BC, Peterson HF, Gelber RD, Goldhirsch A, Gusterson BA, Trihia H, Lindtner J, Cortes-Funes H, Simmoncini E, Byrne MJ, Golouh R, Rudenstam CM, Castinglione-Gertsch M, Allegra CJ and Johnston PG (1997) Prognostic importance of thymidylate synthase expression in early breast cancer. *J Clin Oncol* **15**: 1923–1931
Pinedo HM and Peters GFJ (1988) Fluorouracil: biochemistry and pharmacology. *J Clin Oncol* **6**: 1653–1664
Setälä LP, Kosma V-M, Marin S, Lipponen PK, Eskelinen MJ, Syrjänen KJ and Alhava EM (1996) Prognostic factors in gastric cancer: the value of vascular invasion, mitotic rate and lymphoplasmacytic infiltration. *Br J Cancer* **74**: 766–772
Shiu MH, Perrotti M and Brennan MF (1989) Adenocarcinoma of the stomach: a multivariate analysis of clinical, pathologic and treatment factors. *Hepatogastroenterology* **36**: 7–12
Stelzner S and Emmrich P (1997) The mixed type in Lauren's classification of gastric carcinoma: histologic description and biologic behavior. *Gen Diagn Pathol* **143**: 39–48
Suda Y, Kuwashima Y, Tanaka Y, Uchida K and Akazawa S (1999) Immunohistochemical detection of thymidylate synthase in advanced gastric cancer: a prognostic indicator in patients undergoing gastrectomy followed by adjuvant chemotherapy with 5-fluoropyrimidines. *Anticancer Res* **19**: 805–810
Washtien WL (1984) Increased levels of thymidylate synthetase in cells exposed to 5-fluorouracil. *Mol Pharmacol* **25**: 171–177
Wils J (1996) The treatment of advanced gastric cancer. *Semin Oncol* **23**: 397–406
Yeh K-H, Shun C-T, Chen C-L, Lin J-T, Lee W-J, Lee P-H, Chen Y-C and Cheng A-L (1998) High expression of thymidylate synthase is associated with the drug resistance of gastric carcinoma to high dose 5-fluorouracil-based systemic chemotherapy. *Cancer* **82**: 1626–1631
Yu CC-W, Levison DA, Dunn JA, Ward LC, Demonakou M, Allum WH and Hallisey MT (1995) Pathological prognostic factors in the second British Stomach Cancer Group trial of adjuvant therapy in resectable gastric cancer. *Br J Cancer* **71**: 1106–1110

 *© 2001 Cancer Research Campaign*
